# Epidemiology, ecology and human perceptions of snakebites in a savanna community of northern Ghana

**DOI:** 10.1371/journal.pntd.0007221

**Published:** 2019-08-01

**Authors:** Yahaya Musah, Evans P. K. Ameade, Daniel K. Attuquayefio, Lars H. Holbech

**Affiliations:** 1 Department of Animal Biology and Conservation Science, University of Ghana, Accra, Ghana; 2 Department of Pharmacology, University for Development Studies, Tamale, Ghana; Institut de Recherche pour le Développement, BENIN

## Abstract

**Background:**

Worldwide, snakebite envenomations total ~2.7 million reported cases annually with ~100,000 fatalities. Since 2009, snakebite envenomation has intermittently been classified as a very important ‘neglected tropical disease’ by the World Health Organisation. Despite this emerging awareness, limited efforts have been geared towards addressing the serious public health implications of snakebites, particularly in sub-Saharan Africa, where baseline epidemiological and ecological data remain incomplete. Due to poverty as well as limited infrastructure and public health facilities, people in rural Africa, including Ghana, often have no other choice than to seek treatment from traditional medical practitioners (TMPs). The African ‘snakebite crisis’ is highlighted here using regionally representative complementary data from a community-based epidemiological and ecological study in the savanna zone of northern Ghana.

**Methodology and findings:**

Our baseline study involved two data collection methods in the Savelugu-Nanton District (in 2019 the district was separated into Savelugu and Nanton districts) in northern Ghana, comprising a cross-sectional study of 1,000 residents and 24 TMPs between December 2008 and May 2009. Semi-structured interviews, as well as collection of retrospective snakebite and concurrent rainfall records from the Savelugu-Nanton District Hospital and Ghana Meteorological Authority respectively over 10-years (1999–2008) were used in the study. Variables tested included demography, human activity patterns, seasonality, snake ecology and clinical reports. Complementary data showed higher snakebite prevalence during the rainy season, and a hump-shaped correlation between rainfall intensity and snakebite incidences. Almost 6% of respondents had experienced a personal snakebite, whereas ~60% of respondents had witnessed a total of 799 snakebite cases. Out of a total of 857 reported snakebite cases, 24 (~2.8%) died. The highest snakebite prevalence was recorded for males in the age group 15–44 years during farming activities, with most bites occurring in the leg/foot region. The highest snakebite rate was within farmlands, most severe bites frequently caused by the Carpet viper (*Echis ocellatus*).

**Conclusion:**

The relatively high community-based prevalence of ~6%, and case fatality ratio of ~3%, indicate that snakebites represent an important public health risk in northern Ghana. Based on the high number of respondents and long recording period, we believe these data truly reflect the general situation in the rural northern savanna zone of Ghana and West Africa at large. We recommend increased efforts from both local and international health authorities to address the current snakebite health crisis generally compromising livelihoods and productivity of rural farming communities in West Africa.

## Author summary

Snakebite envenomations cause tens of thousands of deaths and hundred thousands of injuries in many developing tropical countries annually, and sub-Saharan Africa represents an epitome example of this ‘neglected tropical disease’. We here present data collected over a 10-year period applying different methodologies across a typical rural savanna community of northern Ghana. Our data corroborates previous findings from the region that snakebites represent a serious public health threat, and that young and active farmers are particularly at risk, hence compromising both livelihoods and economic wealth of the people. We highlight that many interrelated factors involving both snake ecology and human behaviour in particular, are responsible for the past and current high snakebite prevalence recorded. We conclude that our findings support increased and concerted efforts from both local authorities and state institutions to address the African snakebite crisis that continues unabated. Such interventions require generation of more general baseline data on snake ecology and human behaviour, combined with public awareness campaigns, education and information. To achieve this, we recommend community-based stakeholder meetings involving local people, traditional authorities, as well as public institutions aiming at addressing the ongoing snakebite menace in this part of the world.

## Introduction

Snakebite envenomations constitute one of the most important human-wildlife conflicts, causing considerable yet largely insufficiently known magnitudes of socio-economical losses, morbidity and death [1]. Globally, out of >3,500 snake species, ~600 are venomous, and ~280 are considered medically important, causing a conservatively estimated >1.2 million snakebite envenomations with ~100.000 deaths and >400,000 cases of morbidity annually [1, 2, 3, 4]. Prevailing conservative estimates of the global burden of snakebite envenomations and fatalities are probably highly underrated as majority are based on conventional health facility reports, largely neglecting cases treated by local traditional medical practitioners (TMPs) [1, 5, 6]. Perhaps more realistic annual estimates indicate between 4.5–5.4 million snakebites, 1.8–2.7 million envenomations and up to 138,000 deaths [6, 7]. Snakebite envenomation is largely a disease of poverty, with developing countries in the tropics recording the highest rates of incidence, morbidity and mortality [4, 8, 9]. People engaged in farming, hunting, fishing and other rural activities are at highest risk, mostly bitten on their limbs during work [9, 10]. In many parts of sub-Saharan Africa, the high mortality and morbidity rates are attributed to increased vulnerability caused by both high work risk and exposure to diverse snake habitats, as well as poor infrastructure and limited access to appropriate medical treatment and health facilities [1, 4, 6]. Currently, an estimated ~100 million people, particularly in southeast Asia and Africa, live in vulnerable areas with very high exposure to snake envenomation and lack of effective antivenom therapy [4]. The current ‘global snakebite crisis’ as a disease of poverty, has been misunderstood, underrated, ignored or neglected as a public health issue [5, 8, 11, 12], and has lately regained prominence as one of the most important ‘neglected tropical diseases’ [7].

In order to mitigate the inadequate health care and treatment of victims of snakebite envenomations, concerted international effort is essential to gather steady and inclusive data on the epidemiological nature of snakebites with socio-demographic and geographic dimensions as well as aspects of snake biology and ecology [4, 5, 12, 13]. Mapping of comprehensive datasets is imperative for understanding the dynamics of human-wildlife conflict such as snakebite vulnerability, and constitute the baseline information needed to provide adequate health facilities and supply of antivenom and other therapeutical innovations [1, 2, 6, 14]. However, inclusive community-based information is currently limited from many high-risk areas particularly in sub-Saharan Africa [15, 16]. There is therefore the need for detailed information from studies combining both field and hospital data [4, 6, 9]. Here, we present a comprehensive and complementary epidemiological dataset of snakebite envenomations from northern Ghana, comprising both household and TMP surveys as well as retrospective hospital reports covering the period 1999–2009. Apart from contributing baseline epidemiological snakebite data, our survey, conducted by snake ecologists, also targeted human-wildlife conflict dynamics, with the purpose of providing insight into measures for improving snakebite prevention and facilitation of effective therapeutic methods. We note that the vast majority of snakebite studies are undertaken solely by medical officers, and as such mainly focused on clinical-pharmaceutical, socio-demographic and epidemiological aspects, with much less attention to biology and ecology of humans as well as snakes. An additional objective of this paper was to apply an integrative approach involving snake ecology and common snakebite epidemiology, in order to increase our understanding of the complex human-wildlife conflict that snakebites truly represent. We consider that such holistic research foci are vital and urgently required by international funding agencies and national public health institutions in their joint efforts to address the ongoing global snakebite crisis [2, 4, 7, 10, 12, 13].

## Methods

### Study area

**Fig 1:**
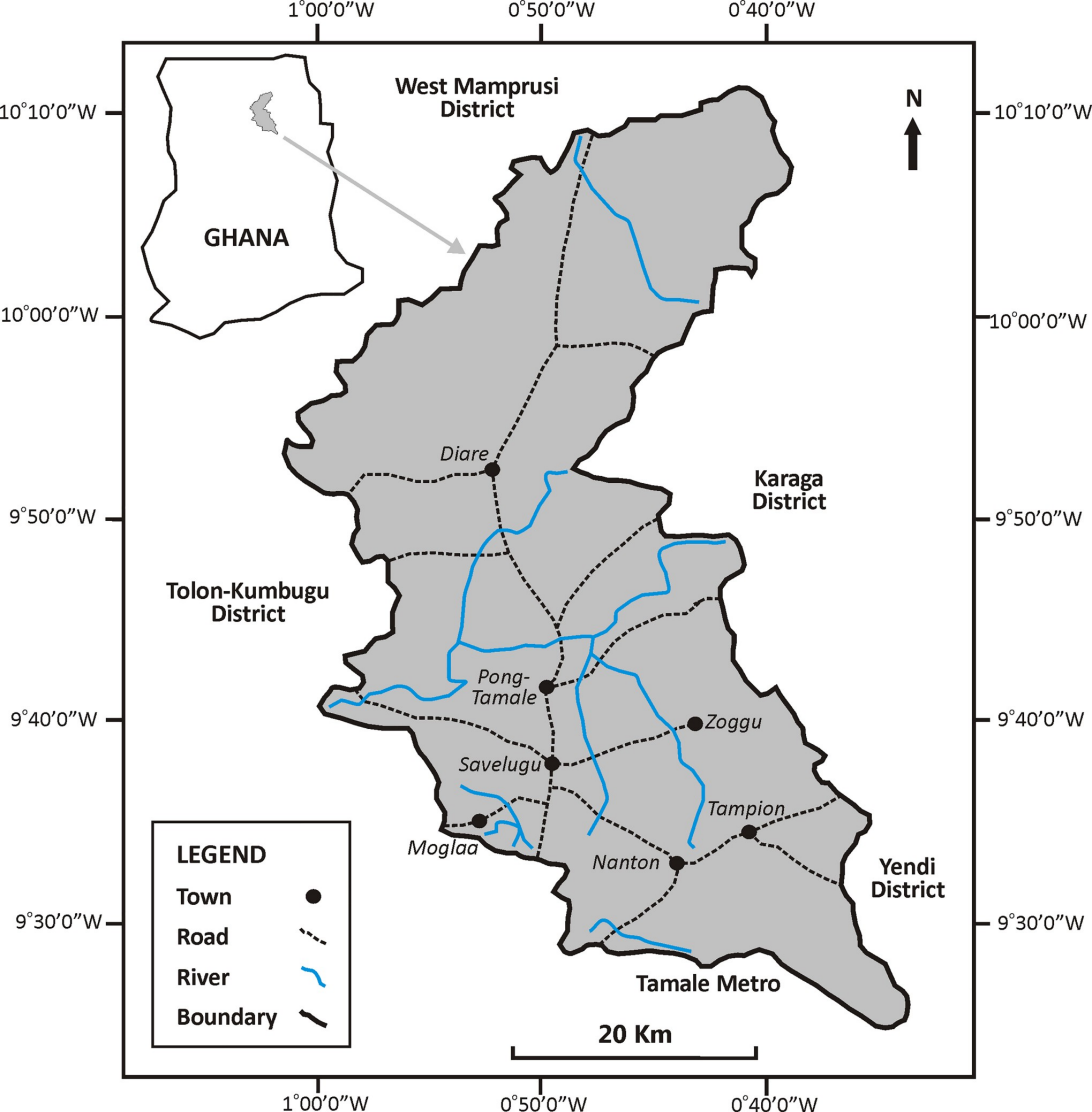
Map of the Savelugu-Nanton District in northern Ghana, showing major roads, rivers, and the seven selected townships for the study. Line drawings produced with CorelDraw version 13.

The Savelugu-Nanton District, covering ~2023 km^2^, is located in the Northern Region of Ghana. It is bordered by five other districts (Fig 1), notably West Mamprusi (north), Karaga (east), Tolon-Kumbungu, and Tamale Metropolitan Area (west) and Yendi (south). Based on a 2010-population census count of 139,283 (female/male %-ratio = 51.5/48.5) and an annual growth rate of about 2.5% for northern Ghana [17], we estimated the total population of the district as ~130,000 in 2008, translating to a mean density of ~64 persons km^-^^2^. The district falls within the Guinea Savanna vegetation zone of northern Ghana, with a single-peak, erratic rainfall pattern, increasing rapidly from April, peaking in August-September, then sharply decreasing during October, and ranging between ~600 to ~1,000 mm annually on average [18]. Mean daily temperatures are usually high, averaging 34°C, with maximum of >42°C and minimum of <15°C [18]. Retrospective monthly rainfall data during 1999–2008 were obtained from the Ghana Meteorological Authority in Accra. The sparsely-populated northern parts of the district have denser vegetation, mostly with regenerating woodlands, compared with the more urbanized south around the Tamale Metropolis characterised by more intensive farming, bush burning and tree felling for charcoal production. Many woodland tree species are drought-resistant and foliage is largely retained during the prolonged dry season (November-March).

### Data collection

#### Ethics Statement

Prior to conduction of interviews, traditional authorities were consulted and appropriate customary rites duly performed with consent of all participants. No ethical clearance was required from any public institution in Ghana for this kind of study, but all patient data was treated with anonymity and permission was granted from hospital authorities at the Savelugu District Hospital in line with codex of conduct.

We conducted a six-month cross-sectional respondent study between December 2008 and May 2009 in the seven most populated communities (= study sites) in the district (Fig 1), comprising 1,000 community residents and 24 TMPs operating in these communities. Both residents and TMPs were subjected to similar, detailed, independently-administered, semi-structured questionnaires. Prior informed consent (PIC) was obtained from traditional authorities in each community after explanation of the scope, purpose and procedure of the study [19]. Such independent administration of interview protocols and PIC enhance the reliability and inclusiveness of respondent information and hence both the quantity and quality of informant data [20, 21]. The selection of residents was stratified across three age-groups, each with gender equality; >30 years (400); 15–30 years (300); <15 years (300). This was attributed to higher encounter frequency of, and more precise and perceptive information gathered from, the elderly. The number of residents sampled from each of the seven study sites generally reflected community size and were; Savelugu (200), Diare and Pong-Tamale (150 each), Nanton, Zoggu, Moglaa and Tampion (125 each). Within each of the seven communities, household units were selected on *ad hoc* basis, inclusively conforming to the gender-age stratification criteria. The selection applied an approach of consecutively enumerating units in a sequence, and adopting an arbitrary criterion of a minimum distance of about 25–50 m and maximum distance of about 50-100m, with longer distances between units in the sparsely populated community fringes, and narrower selection in densely populated community centres. To minimize inconsistencies from recall biases during interviews, the reliability of information and credibility of informants were continually considered. The principal investigator assisted by his local counterpart exercised particular patience and pedagogical skills to explain the topics and agendas involved. The 24 TMPs were selected opportunistically using ‘Snowball sampling’ [19], utilising the confidence gained from one participant to the next, aiming at including nearby as well distant TMPs, in order to minimize selection biases in that regard. Interviews of household respondents (HR) and TMPs aimed at producing three types of data: 1) personal snakebite cases experienced by HR and TMPs; 2) knowledge of snakebite cases of other community members by HR; 3) perception on various variables rather than personal experiences of cases by HR and TMPs. By asking the same questions to both HR/TMPs we were able to test the similarity in responses by using %-ratios for both dichotomous (e.g. male/female, dry/wet season) or polychotonous (e.g. habitat categories for snake encounters, daytime and symptoms) questions. Estimates on derived snakebite rates could then be compared from the two complementary datasets (HR vs. TMPs) across the sections of the communities. As some HR were unable to recall all information pertaining to sub-group variables of each question (e.g. habitat type or symptoms), the %-ratios were based on different totals (n), for which analyses were accounted. Complementing the community-based surveys was a retrospective study of reported cases of snakebites over a 10-year period (1999–2008) that were obtained from the Savelugu-Nanton District Hospital, the largest district hospital that manages snakebite victims. Patient data were analysed anonymously by the lead author and a local assistant, after obtaining formal approval and ethical clearance from the Savelugu-Nanton District Hospital authorities. These data were largely inconsistent and incomplete with other data than date, gender, age and fatalities, so data analysis unfortunately did not include details on treatment, snake culprits and prior TMP consultation. Note that the survey data presented here were parallel to other ecological studies of abundances and distribution of snakes, just as many snake culprits were brought to us for identification. The principal investigator (lead author) identified snakes from secondary data by using local names and using a photo guide of the commonest snakes in the area.

### Data analysis

Frequencies of occurrence (%) in each respondent category were compared among different sub-groups, and associations tested for statistical differences with Fisher’s exact test for 2×2 contingency tables (two sub-groups compared) or χ^2^ *G*-test for three or more sub-groups compared (2×3 contingency tables). Associations between rainfall and snakebite incidences were determined using both GLM and polynomial regression with Pearson’s (*r*) or Spearman rank (*r*_s_) correlation coefficients. To test the similarity in responses (%-ratios only) among various types of data sets (e.g. encounters vs. cases from HR data only, or HR vs. TMP perception data) we used non-parametric Spearman rank (*r*_s_) correlation coefficients and Wilcoxon’s signed rank tests (two-tailed). Whereas retrospective hospital records readily provided estimates of actual annual snakebite incidence and mortality rates per 100,000 inhabitants, the simple interview-based prevalence and case fatality ratios (CFR) expressed as % of totals, only merited such estimates based on particular non-validated assumptions, and therefore reserved for the Discussion only. Hospital and informant data were analysed using Microsoft Excel and GraphPad version 5.01. Significance level was determined at p < 0.05.

## Results

### Household survey

#### Educational level of respondents

Majority (518) of 1,000 respondents (~52%) had no formal education whereas ~44% had only basic primary education (7–9 years of schooling). Only ~4% had additional secondary education (10–12 years of schooling), with <0.5% having tertiary education (>12 years of schooling) only (Table 1). Hence, respondents were primarily made up of either illiterates or people with modest schooling.

**Table 1:** Basic socio-demographic characteristics and snake encounter statistics of the respondent community population (n = 1,000 residents) comprising seven communities surveyed in the Savelugu-Nanton District of northern Ghana (Dec 2008-May 2009).

Respondent variable	No. of respondents (%)
Males	500 (50.0)
Females	500 (50.0)
<15 years old	300 (30.0)
15–30 years old	300 (30.0)
>30 years old	400 (40.0)
No formal education (illiteracy)	518 (51.8)
Basic primary school level (7–9 yrs)	439 (43.9)
Secondary school level (10–12 yrs)	39 (3.9)
Tertiary educational level (> 12 yrs)	4 (0.4)
Experienced a snake encounter	934 (93.4)
Experienced a snakebite personally (E)	58 (5.8)
Witnessed a snakebite (including personal snakebites)*	604 (60.4)
Snakebite incidences witnessed in their community (W)*	799 (79.9)
Total snakebite incidences reported (E+W)	857 (85.7)

*The breakdown of 799 cases witnessed by 604 respondents is found in Table 4.

#### Snakebite prevalence based on personal experience: Comparing gender and age of victims

Considerably more males (~62%) than females (~38%) were reportedly bitten by snakes (n = 58 cases), although the difference was not statistically significant (Fisher’s exact test; p = 0.0778, df = 1), at the 5% level (Table 2). With regards to age, significantly (χ^2^ = 14.616; p = 0.00067, df = 2) more victims were in the age group >30 years (~64%), whereas the group of 15–30 years (~17%) and <15 years (~19%) were similar in snakebite prevalence (Table 2).

**Table 2:** Associations of reported personal snakebite incidences (n = 58) with gender and age-group of 1,000 community members (male/female = 500 each) interviewed in the Savelugu-Nanton District of northern Ghana (Dec 2008-May 2009).

Variable	Sub-group variable	Frequency (%)	Test-statistics	p-value
Gender	Male	36 (62.1)	Fisher’s exact test (2×2)	0.0778
	Female	22 (37.9)		
Age	<15 years	11 (19.0)	χ^2^ *G*-test (2×3) = 14.616	0.00067
	15–30 years	10 (17.2)		
	>30 years	37 (63.8)		

#### Personal encounters with snakes and witnessing of snakebites

As many as 934 (~93%) respondents had encountered snakes during their lifetime and hence been exposed to snakebite risk, and 58 respondents (~6%) claimed to have been bitten by snakes, irrespective of actual envenomation or hospitalisation (Tables 1 and 2). Of the 934 snake encounters (Table 3) majority encountered snakes on their farms (604; ~65%), but also in the bush (~16%) or in their homes (~11%). Considerably lower encounter frequencies were attributed to roads and footpaths (~6%), school facilities (~2%) and open urban drains (<1%). Snake encounters were predominant during afternoon (~50%) and morning (~40%) hours, particularly during the rainy season, accounting for ~72% of yearly records by respondents (Table 3).

**Table 3:** Characteristics of personal snake encounters and witnessing of bites reported (n = 934 encounters and n = 670, 828 or 857 incidences) among 1,000 community members interviewed in the Savelugu-Nanton District of northern Ghana (Dec 2008-May 2009).

Respondent variable	Sub-group variable	Encounters (%)	Bites (%)
Encountering habitat location	Farmlands	604 (64.7)	543 (81.0)
	Bushes	145 (15.5)	56 (8.4)
	House and yards	100 (10.7)	54 (8.1)
	Paths and roads	60 (6.4)	5 (0.7)
	School facilities	19 (2.0)	9 (1.3)
	Open city drains	6 (0.6)	3 (0.5)
Encountering time of the day	Afternoon	471 (50.5)	334 (40.3)
	Morning	372 (39.8)	315 (38.0)
	Evening and night	91 (9.7)	179 (21.6)
Encountering season of the year	Rainy season	669 (71.6)	610 (71.2)
	Dry season	265 (28.4)	247 (28.8)

A total of 604 (~60%) respondents provided knowledge about 799 snakebite cases in total from their respective communities (Tables 3 and 4). Thus, the total number of recorded cases of snakebites, including personal experiences (n = 58) was 799 + 58 = 857, with a total of 24 (CFR ~2.8%) reported deaths (Tables 1 and 5). A total of 13 (~54%) died at hospitals and 11 (~46%) at local TMPs according to the 604 HRs who reported 24 fatalities out of the 857 cases they had witnessed from other community members (W) and experienced (E) personally (E + W = 857; Table 1).

**Table 4:** Snakebite cases reported by each of 604 respondents out of 1,000 community members interviewed in the Savelugu-Nanton District of northern Ghana (Dec 2008-May 2009).

Cases	Respondents	Total
One	449	449
Two	121	242
Three	30	90
Four	3	12
Six	1	6
**Total**	**604**	**799**

**Table 5:** Comparison of actual reported cases and perceptual characteristics of snakebite victims and incidences based on household surveys (HR; *n_max_ = 857 cases) and traditional medical practitioners (TMP; **n = 24 respondents’ perceptions, not cases; ***n = 42 and 65 cases), respectively among 1,000 community members and 24 TMPs, interviewed in the Savelugu-Nanton District of northern Ghana (Dec 2008-May 2009). For perception of best treatment, n* = 1000 cases.

Respondent variable (n = responses)	Sub-group variable	Survey frequency (%)	
		Household	TMP
Gender (*n = 833; **n = 24)	Male	584 (70.1)	22 (91.7)
	Female	249 (29.9)	2 (8.3)
Age-group of victim (*n = 412; **n = 24)	15–30 years	222 (53.9)	13 (54.2)
	>30 years	146 (35.4)	10 (41.6)
	<15 years	44 (10.7)	1 (4.2)
Day time hours of snakebite (*n = 828; **n = 24)	Morning	334 (40.3)	11 (45.8)
	Afternoon	315 (38.0)	10 (41.7)
	Evening and night	179 (21.6)	3 (12.5)
Season of snakebite (*n = 857; **n = 24)	Rainy season	610 (71.2)	20 (83.3)
	Dry season	247 (28.8)	4 (16.7)
Habitat location of snakebite (*n = 670; **n = 24)	Farmlands	543 (81.0)	16 (66.6)
	Bushes	56 (8.4)	7 (29.2)
	House and yards	54 (8.1)	-
	Paths and roads	5 (0.7)	1 (4.2)
	School facilities	9 (1.3)	-
	Open city drains	3 (0.5)	-
Position of snakebite infliction (*n = 828; **n = 24)	Legs and feet	540 (65.2)	19 (79.2)
	Arms and hands	280 (33.8)	5 (20.8)
	Head	7 (0.8)	-
	Trunk	1 (0.1)	-
Symptoms most often reported (*n = 821; ***n = 42)	Swelling	431 (52.5)	14 (33.3)
	Bleeding	320 (38.9)	20 (47.6)
	Local pain	67 (8.2)	-
	Dizziness	3 (0.4)	-
	Blood spitting	-	4 (9.5)
	Sweating	-	2 (4.8)
	Shivering	-	2 (4.8)
Likely causal snake species (***n = 65)	Carpet Viper	n/a	23 (35.4)
	Night Adder		17 (26.2)
	Black-necked Spitting Cobra		13 (20.0)
	Puff Adder/other *Bitis* sp.		8 (12.2)
	Grass snakes/*Psammophis* sp.		4 (6.2)
Treatment methods (*n = 655; **n = 24)	Hospital	392 (59.8)	-
	TMP	262 (40.0)	24 (100.0)
	Self-help	1 (0.2)	-
Preferred treatment	Hospital	798 (79.8)	n/a
(*n = 1,000 respondents)	TMP	196 (19.6)	
	Self-help	6 (0.6)	

*Household survey (HR): For cases where total responses n_tot_ < 857, are explained by failure of respondents to recall details (see Methods).

Of the 857 snakebite victims, majority (~70%) were males, predominantly in the 15–30 year- (~54%) and >30 year- (~35%) age groups (Table 5). Farmlands were the most frequently reported locations (~81.0%) for snakebite, primarily during the rainy season (~71%), in the mornings (~40%) and afternoons (~38%) (Table 5). Snakebites occurred mainly on the extremities, with lower limbs (legs and feet; ~65%) and upper limbs (arms and hands; ~34%) being the most vulnerable, for which swelling (~53%) and bleeding (~39%) were the commonest symptoms reported (Table 5).

The associations between %-respondents for the concurrent data sets of snake encounters versus snakebite experiences showed highly significant positive correlations across the six habitat types (*r*_s_ = 0.9908, p = 0.000127, two-tailed, n = 6,), as well as across the six habitat types, three daytime periods and two seasons combined (*r*_s_ = 0.9545, p < 0.00001, two-tailed, n = 11).

### Traditional medical practitioner (TMP) survey

#### Knowledge and perceptions of snakebite victims and snake culprits

**Fig 2:**
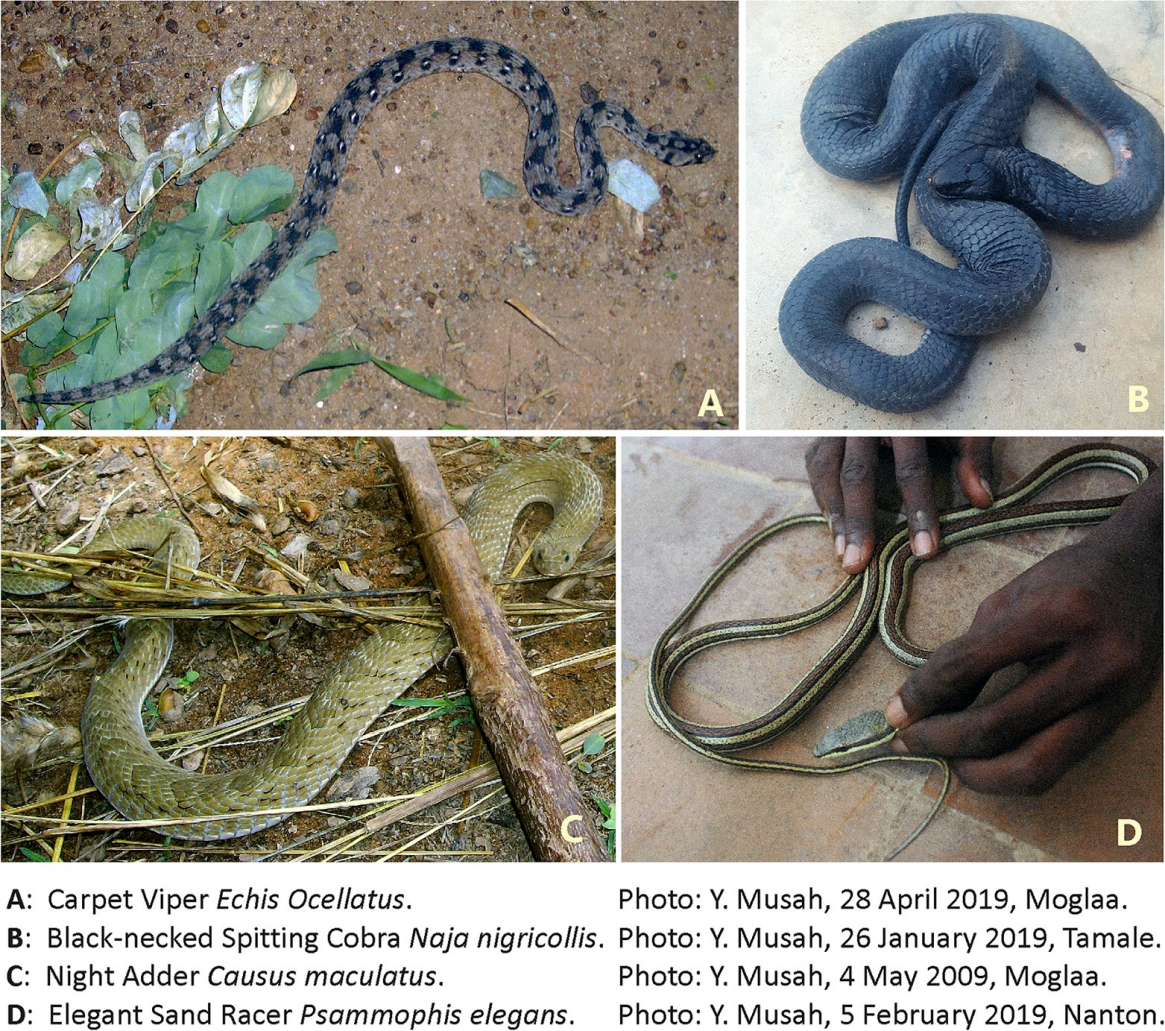
Top-Left (A): Carpet Viper *Echis ocellatus* (Y. Musah, 28 April 2009, Moglaa). Top-Right (B): Black-necked Spitting Cobra *Naja nigricollis* (Y. Musah, 26 January, 2019 Tamale). Down-Left (C): Night Adder *Causus maculatus* (Y. Musah, 4 May 2009, Moglaa). Down-Right (D): Elegant Sand Racer *Psammophis elegans* (Y. Musah, 5 February 2019, Nanton).

From their perceptions (not actual cases), the 24 TMPs reported that majority of patients were males (~92%), in the >30 year- (~54%) and 15–30 year- (~42%) age groups, most often bitten on farmlands (~67%) and bushes (~29%), during the rainy season (~83%), in the morning (~46%) and afternoon (~42%) hours (Table 5). According to the TMPs, most susceptible human body parts are legs and feet (~79%), followed by the arms and hands (~21%), with bleeding (~48%) and swelling (~33%) as most frequently reported symptoms (Table 5). The general snake descriptions provided by the TMPs indicated that three snake species were most likely involved in ~81% of all cases reported (Fig 2); Carpet Viper *Echis ocellatus* (~35%), Night Adder *Causus maculatus* (~26%), and Black-necked Spitting Cobra *Naja nigricollis* (~20%). The %-responses to 27 similar questions (i.e. the first 27 sub-group variables = rows tested pair wise in Table 5) among 857 HRs matched well with those %-responses of the 24 TMPs (Table 5), as shown by the two datasets not being significantly different (*r*_s_ = 0.8469, *r* = 0.9245, p < 0.00001, two-tailed, n = 27; z = 0.0721, p = 0.9442).

### Retrospective hospital study

#### Snakebite prevalence: comparing gender and age of victims

Based on the 10-year (1999–2008) retrospective records of snakebite cases (n = 450) at the main district hospital, significantly more males, particularly in the 15–44 years age group (χ^2^ *G*-test = 29.56, p < 0.00001, df = 2), were bitten than females, as well as males in the <15 year- and >44 year- age groups (Table 6).

**Table 6:** Statistically-tested associations of snakebite cases (n = 450) with gender and age-group reported at the Savelugu-Nanton District Hospital in northern Ghana (1999–2008).

Sub-group variable	Gender		Total (%)
Age group (years)	Males (%)	Females (%)	
<15	90 (20.0)	12 (2.7)	102 (22.7)
15–44	133 (29.6)	91 (20.2)	224 (49.8)
>44	73 (16.2)	51 (11.3)	124 (27.5)
Total	296 (65.8)	154 (34.2)	450 (100.0)
Statistical Test: χ^2^ *G*-test (2×3) = 29.56, p < 0.00001, df = 2			

Across all three age groups, males dominated in number of reported cases, whereas the females constituted relatively fewer cases in the youngest age group (2.7%) but 4–8 times more in the two older groups (Table 6). For both males and females, the 15–44 years group dominated in almost 50% of all cases reported. These results are largely consistent with age-gender trends of personally experienced snakebite cases (n = 58) reported among 1,000 respondents in the 2008–2009 household survey, with dominance of males across all age groups, and in particular for the >30 year age group (Table 2). In summary, the most and least vulnerable to snakebites were young to middle aged men and younger girls respectively. Out of the 450 recorded snakebite cases only one death was recorded, producing a CFR per 10 year of 1/450 = 0.22%, and thus an annual CFR = 0.022%.

### Correlations between rainfall and snakebites for the period 1999–2008

**Fig 3:**
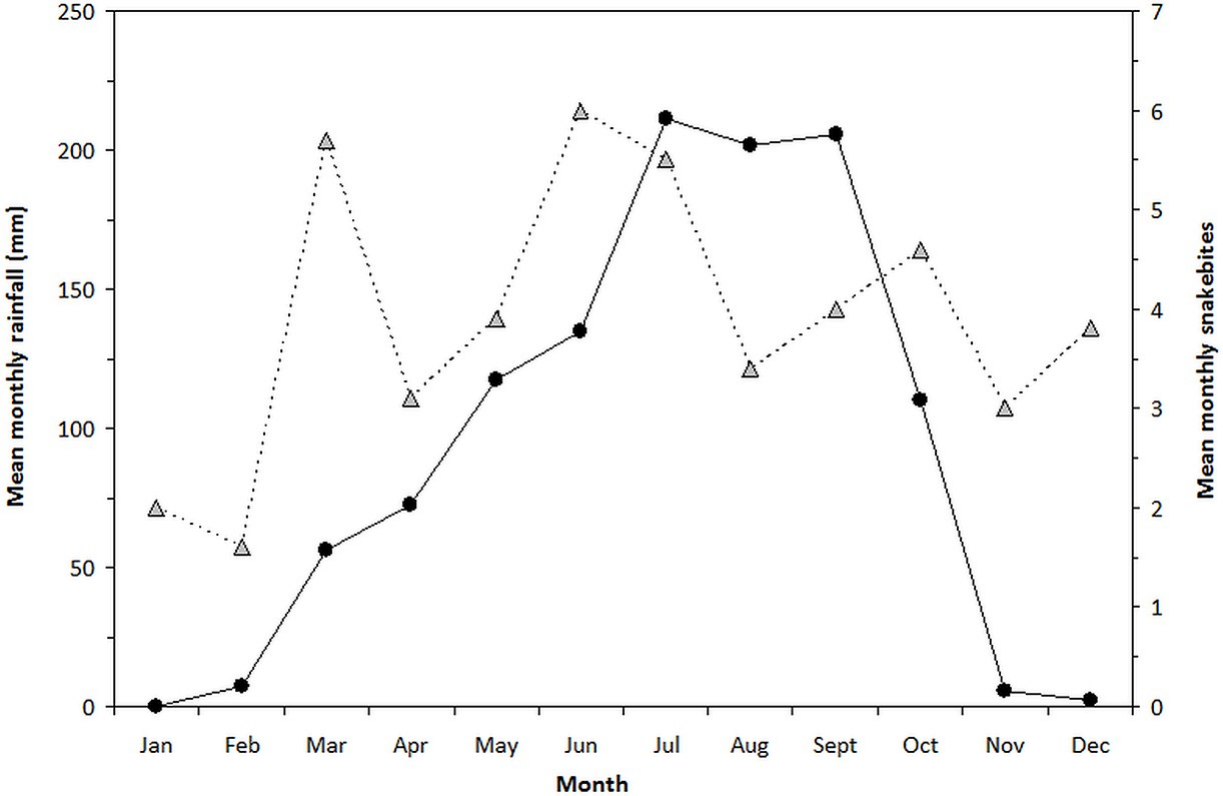
Mean monthly precipitation (black circles and solid line) and mean monthly number of snakebite incidences (grey triangles and broken line) recorded at the Savelugu District Hospital (1999–2008).

**Fig 4:**
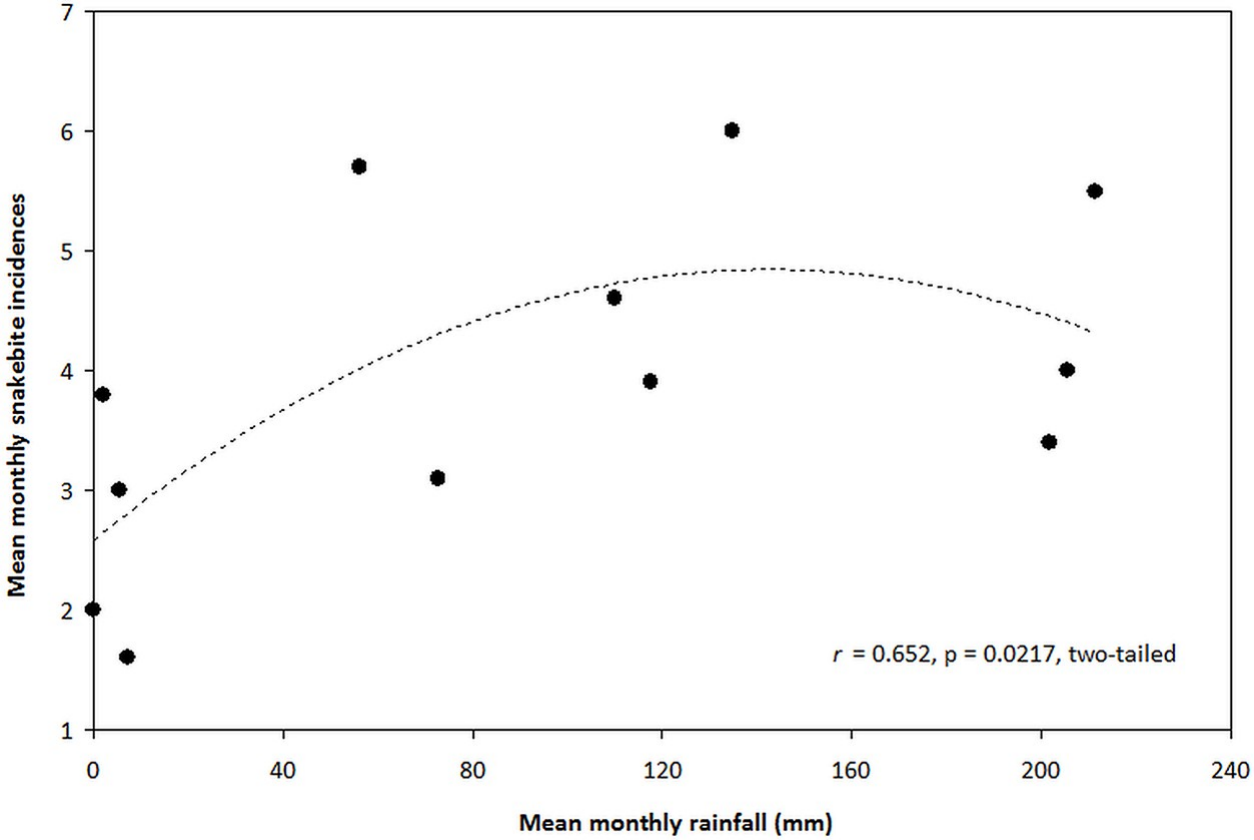
Correlation between mean monthly precipitation and mean monthly number of snakebite incidences recorded at the Savelugu District Hospital (1999–2008).

By comparing concurrent data sets of mean monthly rainfall with monthly records of snakebites reported to the district hospital in 1999–2008 (Fig 3), we were able to evaluate the perception reported among community members (HRs and TMPs) that snakebites were more prevalent during the rainy season (Tables 3 and 5). The 10-year plot of rainfall-snakebite monthly means depicted a clear unimodal rainfall curve with a single peak from July to September, whereas snakebite incidences displayed a trimodal pattern with peaks in March, June-July and October (Fig 3). Hence, the largely overlapping rainfall-snakebite trend demonstrated a positive association between rainfall and snakebite frequency, in line with respondent (both HR victims/witnessing and TMPs) perceptions (Tables 3 and 5). We tested this apparent positive association by plotting 10-year pair-wise data of mean monthly rainfall with mean monthly snakebite cases, and found a weak statistically significant (*r*_s_ = 0.587, p = 0.0446, two-tailed, n = 12) positive linear correlation between the two variables (Fig 4). However, the correlation for GLM-regression using Pearson’s correlation coefficient was not significant (*r* = 0.521, p = 0.0822, two-tailed, n = 12). We also performed a 2^nd^ degree polynomial regression which showed a higher level of significance (*r* = 0.652, p = 0.022, two-tailed, n = 12) compared with the GLM-regression, thereby indicating a hump-shaped association pattern (Fig 4). Thus, although snakebite rates are generally linked to rainfall levels, extreme amounts of rain appear to reduce this positive correlation.

## Discussion

Our study applied a regionally representative set of complementary epidemiological data from different community sectors for northern Ghana, by targeting various demographic sub-groups as well as both traditional and conventional management of snake envenomations at the district level. With a relatively large sample size of 1,024 respondents (~0.8% of district level population) covering both dry and wet seasons, and a retrospective 10-year bivariate data sample of snakebite incidences and rainfall patterns, our study represents an important contribution to epidemiological snakebite studies for this typical West African savanna region. Previous epidemiological studies have largely been limited to short-term data series with smaller sample sizes of predominantly retrospective hospital records for Ghana [22, 23, 24] and sub-Saharan Africa [25, 26, 27, 28, 29], where complementary or nationwide long-term published studies remain scarce [1, 16, 30, 31]. Estimates of regional or national snakebite burdens based exclusively on hospital records inevitably neglect many snakebite cases treated at home or by TMPs [1, 4, 16, 30]. In contrast, our study provided a rare opportunity to test consistencies in epidemiological baseline information gathered from different sections of a typical rural savanna community in West Africa [16], regarding general demography and human-snake conflict, as well as snake toxicology and ecology. In order to enhance advances in public health capacity to address snakebite envenomation, it is important to provide inclusive and holistic evaluations of implicating factors across the whole community and over long periods of time, including ecological aspects [2, 4, 7, 30]. Specifically, it is important to ask where, when and why snakebites occur, and to identify and evaluate pertinent factors that preclude or facilitate their occurrence, treatment and prevention [1, 4, 10, 12].

We recognise that despite a relative to the small sample area (~2,023 km^2^) large sample size of complementary data, biases are inevitable from the study design as well as sample procedures and protocols. We are also aware that randomized standard sociological census procedures were not adhered to, but we truly believe that by spreading sampling across seven distinct townships including both outskirts and centres enabled us to get a good representative sample, emphasising strict adherence to age-gender stratification criteria. Some margin of recall bias in ability was acceptable based on a general assumption that 10 years remembrance can only be completely accurate in few cases. In that context, we found high consistencies of responses both within and between our complementary data set, indicating that conclusions drawn from both datasets (e.g. HR vs. TMP) are similar, and the information most likely reliable for fair generalisations. The comparison of snake encounters and cases based on HR data likewise suggests that an increase in snake encounters results in increased snakebites, and the implication is that snake encounters represent a crude index of snake-human conflict. We also recognise that our CFR is probably underestimated due to the fact that the 799 witnessed cases could be shared among independently-selected respondents, hence the 24/857 = 2.8% would be higher accordingly. We however have no means of assessing that potential bias but if say 10% of the 799 cases are overlapping, then it will result in a CFR = 3.1%, and if 50% shared, CFR = 5.2%.

Our study confirms the general assertion that snake envenomations leading to significant levels of morbidity and mortality are symptomatic of rural deprivation and poverty in sub-Saharan Africa, where educational levels are pronouncedly low within farming communities subject to limited infrastructure and mobility [4, 8, 9, 12]. Our respondents were primarily either illiterates or people with modest schooling, a demographic feature characteristic of the mostly deprived rural areas of northern Ghana, and a tendency particularly pronounced among women and the elderly. We located 24 TMPs in seven larger communities, demonstrating that the TMP-inhabitant ratio of at least 1:5,000 (~24/130,000) is ~10 times lower than the regional doctor-patient ratio for northern Ghana in 2009 [32]. As the road network in the Savelugu-Nanton District was fairly well developed during our study, we attribute the high patronage of TMPs mainly to low educational level and income, limited access to orthodox medical facilities as well as high costs or lack of sufficient and effective antivenom administration. This TMP-seeking behaviour is widespread in Asia [33] and Africa [16, 34, 35, 36] where herbal extracts of diverse medicinal plant assemblages [37] are often either culturally preferred or are the only affordable or alternative therapeutic treatment available among low-income rural communities with high levels of illiteracy and strong superstitious beliefs [16, 19, 38, 39, 40, 41]. Recent newspaper articles in Ghana indicate that this situation has not improved and probably even aggravated lately [42, 43].

Based on the 1,000-respondent household survey, we conservatively estimated a snakebite prevalence of ~6% (58 personal cases out of 1,000), and a CFR of ~3% (24 out of 857 personal/witnessed cases; 2.8%). This translates to 58×130 equivalent to 7,540 snakebite incidences and 7,540×0.028 equivalent to ~210 deaths at the district level, for a total district population estimated at ~130,000 in 2008. In comparison, a CFR of 1% was found in a similar community-based study in Senegal [16]. We recognise that our incidence and mortality data are based on unknown individual informant recollection periods, and we can therefore not explicitly translate these district level figures into annual snakebite and mortality rates. However, assuming a recollection period of about 10 years retrospectively for each HR respondent on average (n = 1,000), the estimated annual snakebite cases and fatalities at district level (population of 130,000) may probably reach 7540/10 = 754, equivalent to 754/1.3 = 580 per 100,000 per year or ~0.58%, and 210/10 = 21, equivalent to 21/1.3 ~ 16 per 100,000 per year or ~0.016% respectively. A longer average respondent recollection period is unlikely, as both young and old respondents exhibited limited memory beyond 10 years. Moreover, if such a period was ~5 years, it would translate to a doubling of cases and fatalities, which likewise appears unlikely. The mean monthly number of cases reported to the largest district hospital was 3.9 over the 10-year period (1999–2008), which equals an average of 46.6 cases yearly (Fig 3). Based on hospital records, the whole district therefore probably has a burden of at least 50 reported cases yearly, and will probably double if the reporting percentage is up to 50% as estimated in similar studies [12, 16, 41]. In this context, we found that out of 655 cases witnessed by HRs, as much as 40% were claimed to have been treated by TMPs, and 60% at the hospital, which is close to the 50–50% reporting ratio found in similar studies [12, 16, 41]. Our upper-lower limits of annual snakebite envenomations during the 10-year period therefore range from ~100–750 per 130,000 (2008-population), equivalent to ~77–580 per 100,000 inhabitants (~0.08–0.6%), with a mortality rate of ~2–16 per 100,000 (~0.002–0.016%). The population of Savelugu-Nanton District in 2008 was ~130,000, constituting ~6% of the population in the Northern Region of Ghana [17], so we conservatively estimate about 1,700–12,500 envenomations with about 50–350 fatalities annually (CFR ~3%) in the northern part of the country with a 2008 population of ~2.2 million. This estimated range of fatalities constitutes 20–95% of a Ghana national estimate of about 250–375 [40], and is within the range of current available estimates for the sub-region [1, 9] and other estimates from northern [23] and central [22, 44] Ghana, but 5–10 times higher than southwest Ghana [24]. Our conservative estimates also indicate that in the Northern Region (NR) of Ghana, the approximate requirement of AV snakebite treatment ranges from 500–600 units annually (not vials) per 100,000 inhabitants. With an approximate 2019-population in the NR of ~3 million, this estimate translates to 15,000–18,000 treatment units annually.

The human deprivation, psychosocial despair, and loss of income resulting from snakebite morbidity and mortality [7, 35, 40], are clearly highlighted by our complementary data from the northern part of the savanna zone in Ghana, where annual snakebite burdens are the most severe across the entire country [45]. Our data also suggest that a large proportion (40–85%,based on estimates from hospital records versus HR data = 650/750 ~ 85%; actual percentage from 655 cases reported ~40%) of snakebites are never reported to conventional medical treatment facilities but managed by TMPs, treated at home treatment or left untreated, often with fatal consequences [16, 24, 46]. Long delays in antivenom treatment often lead to severe cases of morbidity (e.g. amputations) or death, even if such treatment is administered correctly [22, 44, 47, 48]. Our data support this assertion as the 10-year hospital records revealed a CFR of 1/450 = 0.22% equivalent to 0.022% per year, in relation to the 2.8% CFR reported from HR data, of which 60% died at the hospital, probably due to delays and prior TMP consultation. Reportedly, some severe cases brought to the hospital were never recorded if patients died upon arrival, and hence never admitted and treated. It is therefore important to ascertain the extent of both untreated and delayed treatments in order to address these clinical and epidemiological shortcomings in the management of snakebites in remote rural parts of Ghana and sub-Saharan Africa [2, 13, 15, 40, 44]. We hope our snakebite data analyses from the Savelugu-Nanton District, a typical northern savanna community in Ghana, will aid in this direction.

We consistently found that young to middle-aged males (age group = 15–44 years) were most at risk of snakebites, corroborating similar findings from Ghana [22, 23, 24], other parts of West Africa [15, 25, 26, 27, 47, 49, 50] and Africa in general [30, 34, 51, 52, 53]. Adolescent and young men in their twenties are among the most active and adventurous, albeit least cautious, section of rural African people [24, 27, 47, 54]. They expose themselves to snake encounters by risky behaviour during land clearing, harvesting, bush and charcoal burning, hunting and commuting on foot during dark or early morning hours with impaired visibility in dim light and dense vegetation [24, 50, 53]. Minors and the elderly who are traditionally home-bound, are less involved in farming activity, and are generally more cautious in their behaviour, which possibly explains their much lower snakebite vulnerability [31]. Some girls and the elderly were however bitten at home or in school facilities, indicating that everyone is at risk of snakebites [7, 24, 31, 46, 54, 55]. Contrary to our findings, some studies in Africa actually report relatively higher snakebite prevalence in children, including girls, probably as a result of higher child-labour engagement or poorer parental care and supervision in such areas [23, 31, 53, 54, 56, 57, 58, 59].

Our data from HRs and TMPs indicated that farmlands and bushes, and to a lesser extent, residential areas and roads are typical habitats for snakebite, as corroborated by several other studies in rural Africa [16, 31, 38, 53, 54]. Likewise, majority of bites occur during the peak farming periods (e.g. shea nut, millet and yam) when land is prepared for planting (March-April), maintained by weeding (May-June), and harvested (June-October) [23, 50, 54]. Our long-term bivariate rainfall-snakebite data indirectly reflect the strong link between peak seasons of farming and rainfall, partly because artificial water sources are largely unavailable to the small-scale peasant farmer in northern Ghana and other parts of arid savanna zones in Africa. However, even though rainfall and snakebites are generally positively-correlated, our data also suggest that very intense rainfall reverses the trend, possibly caused by decreased activity of both snakes and humans as well as lower snakebite reporting rates due to the reduced mobility from flooding and erosion of basic road infrastructure. Although the dry season recorded lower snakebite incidences, farmers are also at risk during this period, often being bitten during bush or charcoal burning, hunting, harvesting firewood, and at home during reduced farming activity in November-April [22, 50, 53].

As agricultural mechanisation is particularly low in northern Ghana, predominantly manual activities using the limbs expose farmers to snakes concealed in vegetation, soil and crops. This may explain why those extremities are the most prone to snakebites [23, 47, 50, 54]. Farming activities primarily take place during morning or late afternoon in order to avoid excessive sun exposure and heat during midday. Such factors also result in the poikilothermic snakes seeking warm areas during morning hours and shade in the hot midday and early afternoons. Nocturnal snakebites were mostly confined to homes and roads, a pattern found in many other rural areas of Africa, and attributed to walking barefoot, or inappropriate footwear and poor domestic lighting conditions [25, 31, 48, 54].

Snakebite prevalence, risk and rates are determined by several interrelated factors pertaining to both snakes and humans, notably the density, activity and behaviour of the snake culprits and their victims. Snake density appears the most important factor, as the commonest species are also often the most prevalent as culprits [23, 49]. Snake density is related to habitat type and human disturbance, as well as food sources and hiding places. It is also inversely related to human density in many parts of sub-Saharan Africa [27, 30, 49], and therefore directly related to both destruction of suitable habitats as well as human persecution, especially in most African cultures with great dislike of snakes. Snakebite prevalence is lower in urban centres devoid of natural vegetation, and where snakes are at higher risk of being detected and killed [30]. Our data were in line with this inverse human-snake density-relationship, as evidenced by lower snakebite incidences in the more densely populated communities (e.g. Savelugu and Nanton) closer to Tamale, the regional capital with >300,000 inhabitants.

Apart from densities of both humans and snakes, activity patterns and behaviour are two other important factors highly correlated with habitats, daytime hours and season. During rains, in which farming activities peak in the savanna zone, snakes often synchronise their breeding periods with high prey abundances [60, 61]. Likewise, density and activity of prey such as amphibians, birds, lizards, murid rodents and other snakes increase during rains, due to prey availability (mainly invertebrates) increasing along with the flourishing vegetation. The number of active farmers, combined with actively-hunting and breeding snakes tends to increase human-snake encounters, often resulting in bites and envenomations [49]. Similarly, the density-activity influence on human-snake encounters and the behaviour of the snake culprits as well as victims are important factors. Risky behaviour during farming, hunting and firewood collection (e.g. using unprotected limbs to grasp, cut, dig, lift and pick tools, foods and other important farm-related items), increases the probability of unintendedly provoking and even attacking snakes [31, 54]. In response, the disturbed snakes may vigorously defend themselves either by hissing, inflating hoods, whipping tails, venom spitting or biting. Certain species appear more docile and reluctant to strike even if trodden, such as Gaboon Viper *Bitis gabonica* or grass snakes/sand racers (e.g. *Psammophis elegans*) while others tend to be extremely aggressive and prone to strike at the least provocation or threat such as Black-necked Spitting Cobra *Naja nigricollis* and Carpet Viper *Echis ocellatus* (Fig 2). Some species retreat long before humans are even close, whereas others stay put and motionless until it is too late to detect their presence before they strike. Highly mobile species may enter houses, garages and even cars in search of prey or warm places (e.g. cobras and small vipers), while other slow-moving species (e.g. large vipers) avoid such human-trafficked areas which pose great human-detection risks. As in the majority of West African studies, and as recorded from all sections of our study population, three snake species were by far the commonest snakebite culprits; Carpet Viper, Night Adder *Causus maculatus* and Black-necked Spitting Cobra [16, 23, 26, 35, 40, 46, 48, 49]. These three species were also among the commonest found in our study area, as shown by extensive ecological censuses [62]. Polyvalent antivenom of these species in particular, is therefore essential in this part of northern Ghana [22, 23, 44].

## Conclusion

Our community-based complementary data clearly demonstrate the gravity of snakebite envenomations in the savanna zone of northern Ghana, and the relatively high burden of incidence, morbidity and mortality. These findings cannot be overemphasised with respect to their negative implications for public health, agricultural productivity, social welfare and economic growth. In order to address the shortcomings of adequately-trained personnel, effective treatment with antivenom, and other efficient therapy, we recommend concerted efforts from both conventional and TMPs to monitor, map and analyse snakebite incidences at district levels, including scientific efficacy testing of complementary therapy to expensive and unavailable polyvalent antivenoms. We also conclude that unnecessary delays at TMPs often result in futility and complicates subsequent conventional hospital treatment, hence the need to improve infrastructure, information on risks associated with TMP treatment, and availability of effective polyvalent AV therapy (e.g FAV-Africa). We recommend that each district hospital in the NR of Ghana has a capacity of treating 5–600 snakebites annually per 100,000 inhabitants, and a total treatment capacity in the NR of maximum 20,000 cases annually. Additionally, community members, particularly the youth, should be sensitised on risky snakebite behaviour, snake biology and measures to prevent and minimise the likelihood of snakebites. Such interventions could be achieved through community meetings, education and awareness sessions, and steady contact in the field with public health authorities liaising with traditional rulers and TMPs.
